# Supplementary material for: Differential meta-analysis of RNA-seq data from multiple studies
Source: BMC Bioinformatics. 2014 Mar 29;15:91. doi: 10.1186/1471-2105-15-91 (PMC4021464; doi:10.1186/1471-2105-15-91)
Supplement: Additional file 1 — Supplementary materials. This document contains a discussion concerning the filtering of RNA-seq data, supplementary information about the characteristics of the data and the Ingenuity Pathways Analysis discussed in the real data analysis, and supplementary figures. [file 1471-2105-15-91-S1.pdf]

Supplementary Materials: Differential meta-analysis of RNA-seq data from multiple studies

A. Rau[*†] G. Marot[‡§] F. Jaffrézic[*†]

Contents

1 Filtering RNA-seq data using *HTSFilter* **1**

2 Data characteristics **2**

3 Top biological functions found with Ingenuity Pathways Analysis **3**

4 Supplementary Figures **4**

1 Filtering RNA-seq data using *HTSFilter*

In this work, we filter weakly expressed genes using the *HTSFilter* Bioconductor package, which implements a data-based filtering procedure based on the calculation of a global Jaccard similarity index among biological replicates for read counts arising from replicated transcriptome sequencing (RNA-seq) data; see Rau et al. (2013) and the *HTSFilter* vignette for additional details. This technique provides an intuitive data-driven way to filter RNA-seq data and to effectively remove those genes that contribute to a peak of raw p-values close to 1, due to the discretization of p-values from conditional tests (such as the Fisher's exact test) for small counts. This latter point is particularly important for the p-value combination methods (Inverse Normal and Fisher) investigated in the main paper, as both rely on an assumption of uniformly distributed p-values under the null hypothesis. Briefly, the *HTSFilter* method seeks to identify the threshold that maximizes the filtering similarity among replicates (as measured by the Jaccard similarity index), that is, one where most genes tend to either have normalized counts less than or equal to the cutoff in all samples (i.e., filtered genes) or greater than the cutoff in all samples (i.e., non-filtered genes). The data-based filter is chosen by examining the behavior of the global Jaccard index (see Supplementary Figure 10), and identifying the cutoff that corresponds to the maximum global Jaccard index.

For both the real and simulated data, we note that for individual per-study analyses of differential expression (and consequently, for the p-value combination methods), data filters are applied independently to each study (e.g., the left and middle panels of Supplementary Figure 10) following estimation of library sizes and dispersion parameters, meaning that it is possible for a gene to be filtered in one study and not in another. For the DESeq approaches, both with and without a fixed study effect, a data filter is applied to all studies simultaneously (e.g., right panel of Supplementary Figure 10) following estimation of library sizes and dispersion parameters. To apply the filter, genes

[*]INRA, UMR1313 Génétique animale et biologie intégrative, 78352 Jouy-en-Josas, France
[†]AgroParisTech, UMR1313 Génétique animale et biologie intégrative, 75231 Paris 05, France
[‡]Université Lille Nord de France, UDSL, EA2694 Biostatistics
[§]Inria Lille Nord Europe, MODAL

with maximum normalized counts less than the previously identified data-based thresholds are subsequently filtered from the analysis (and thus, identified as non-differentially expressed).

2 Data characteristics

Supplementary Table 1: Data characteristics for each sample in the *H. sapiens* data of Strub *et al.* (2011), including the total number of reads, the number of unique reads, and the former divided by the latter.

Study	Condition	Replicate	Total # of reads	# of unique reads	Total / unique
1	siMTF	1	27,193,572	7,468,919	3.64
1	siMTF	2	35,606,942	6,227,177	5.72
1	siLuc	1	30,891,601	8,326,707	3.71
1	siLuc	2	30,455,011	3,121,413	9.76
1	siMTF	3	28,692,182	4,719,313	6.08
1	siLuc	3	31,580,414	6,556,961	4.82
2	siLuc	1	22,659,403	13,010,902	1.74
2	siLuc	2	26,028,695	15,921,568	1.63
2	siMTF	1	24,988,432	14,602,944	1.71
2	siMTF	2	25,688,964	15,323,516	1.68

3 Top biological functions found with Ingenuity Pathways Analysis

Supplementary Table 2: Top five biological functions found with Ingenuity Pathways Analysis (Ingenuity® Systems, www.ingenuity.com) for the list of genes uniquely identified by the Fisher p-value combination method, with respect to the global analysis with fixed study effect.

Name	p-value	#Molecules
Cancer	9.49E-04 - 3.08E-02	152
Connective Tissue Disorders	9.49E-04 - 3.08E-02	20
Developmental Disorder	9.49E-04 - 3.08E-02	84
Gastrointestinal Disease	9.49E-04 - 3.08E-02	42
Hepatic System Disease	9.49E-04 - 3.08E-02	30

Supplementary Table 3: Top five biological functions found with Ingenuity Pathways Analysis (Ingenuity® Systems, www.ingenuity.com) for the list of genes uniquely identified by the global analysis with fixed study effect, as compared to the Fisher p-value combination method.

Name	p-value	#Molecules
Organismal Injury and Abnormalities	7.44E-06 - 2.17E-02	43
Connective Tissue Disorders	8.05E-06 - 2.17E-02	27
Dermatological Diseases and Conditions	8.05E-06 - 2.17E-02	26
Developmental Disorder	8.05E-06 - 2.17E-02	44
Hereditary Disorder	8.05E-06 - 2.17E-02	45

Supplementary Table 4: Top five biological functions with Ingenuity Pathways Analysis (Ingenuity® Systems, www.ingenuity.com) for the list of genes found in common between the Fisher p-value combination method and the global analysis with fixed study effect.

Name	p-value	#Molecules
Cancer	1.41E-15 - 4.07E-04	1541
Reproductive System Disease	6.47E-13 - 3.98E-04	527
Gastrointestinal Disease	1.41E-12 - 2.96E-04	485
Hematological Disease	2.08E-10 - 2.71E-04	244
Neurological Disease	2.61E-09 - 3.41E-04	530

4 Supplementary Figures

Supplementary Table 5: List of Supplementary Figures included in this document.

Figure	Page	Caption
1	5	Area under the curve (AUC) of the Receiver Operating Characteristic (ROC) curve for all simulation settings.
2	6	False discovery rates (FDR) for all simulation settings.
3	7	Integration-driven discovery rates (IDR) for all simulation settings.
4	8	Integration-driven revision rates (IRR) for all simulation settings.
5	9	Receiver Operating Characteristic (ROC) curves, for the simulation settings not demonstrated in the main paper.
6	10	Sensitivity, for the simulation settings not demonstrated in the main paper.
7	11	Proportion of true positives among unique discoveries for DESeq with a fixed study effect (orange bars) and Fisher (red bars) for the simulation settings not demonstrated in the main paper.
8	12	Per-gene dispersion parameter estimates from *DESeq* plotted against mean normalized counts, calculated for Study A independently (left), Study B independently (middle), and Studies A and B together (right), with fitted gamma regressions superimposed as red lines.
9	12	Inter-study variability calculated on the Strub *et al.* data, with a $\mathcal{N}(0, \sigma = 0.5)$ density superimposed in blue.
10	13	Global Jaccard index calculated for a variety of threshold values for Study A independently (left), Study B independently (middle), and Studies A and B together (right), with a loess curve (blue line) superposed and data-based threshold value indicated by the red cross and red dotted line.
11	13	-Log$_2$ raw p-values in Studies A and B for genes identified as differentially expressed using the Inverse normal (left) and Fisher (right) p-value combination methods, after adjusting combined p-values to control the false discovery rate at level $\alpha = 0.05$. Genes with differential expression conflicts are identified with red points.
12	14	-Log$_2$ raw p-values in Studies A and B for genes identified as differentially expressed using the Inverse normal (left) and Fisher (right) p-value combination methods, after adjusting combined p-values to control the false discovery rate at level $\alpha = 0.05$. The black dotted line represents the diagonal, and the red line represents a loess curve.

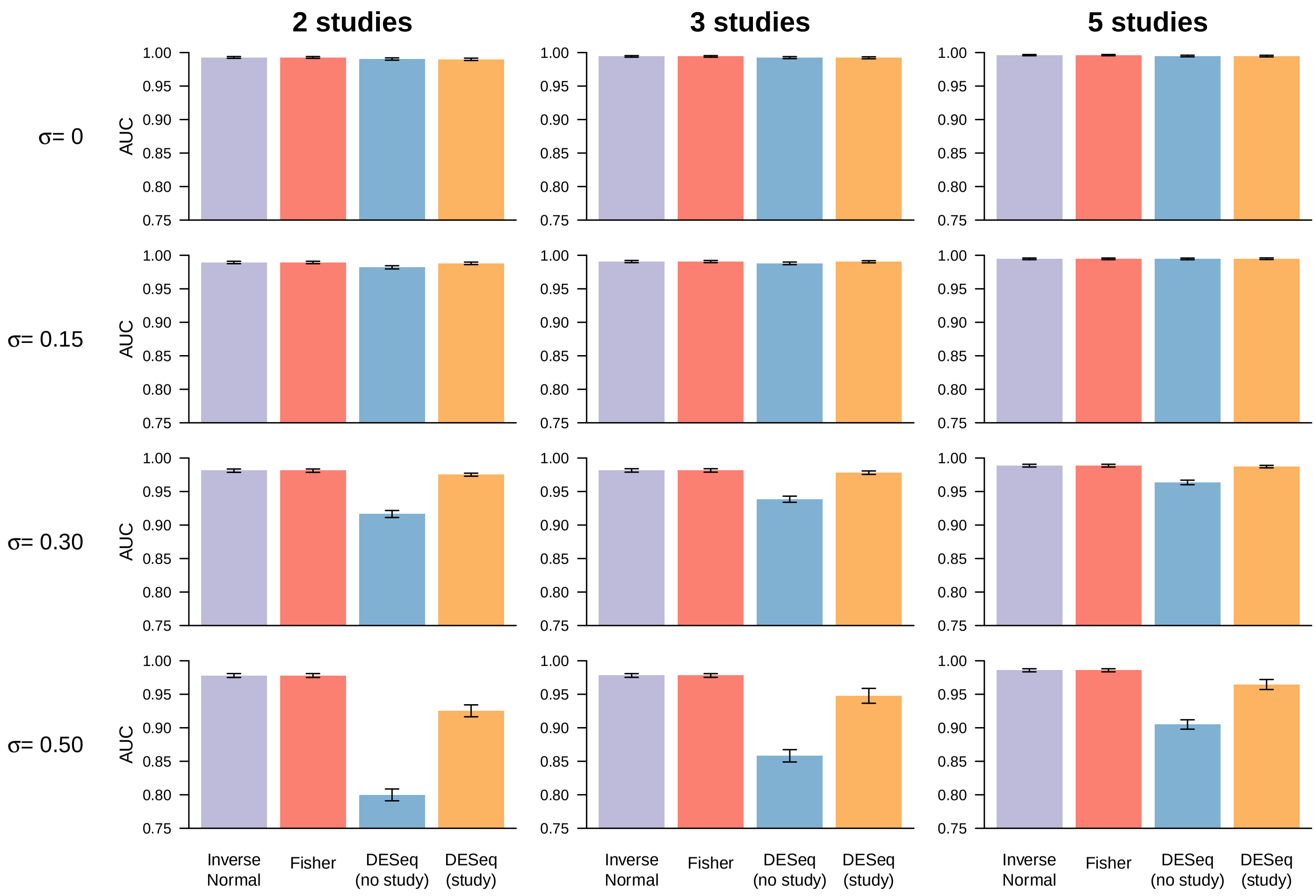

Supplementary Figure 1: Area under the curve (AUC) of the Receiver Operating Characteristic (ROC) curve for all simulation settings. Each barplot represents the results of a particular setting, with columns corresponding (from left to right) to simulations including 2 studies, 3 studies, and 5 studies, and rows corresponding (from top to bottom) to simulations with inter-study variability set to $\sigma = 0$, $\sigma = 0.15$, $\sigma = 0.30$, and $\sigma = 0.50$ (no inter-study variability to large inter-study variability). Within each barplot, from left to right: Inverse Normal (purple bars), Fisher (red bars), DESeq with no study effect (blue bars), and DESeq with a fixed study effect (orange bars). Error bars represent one standard deviation.

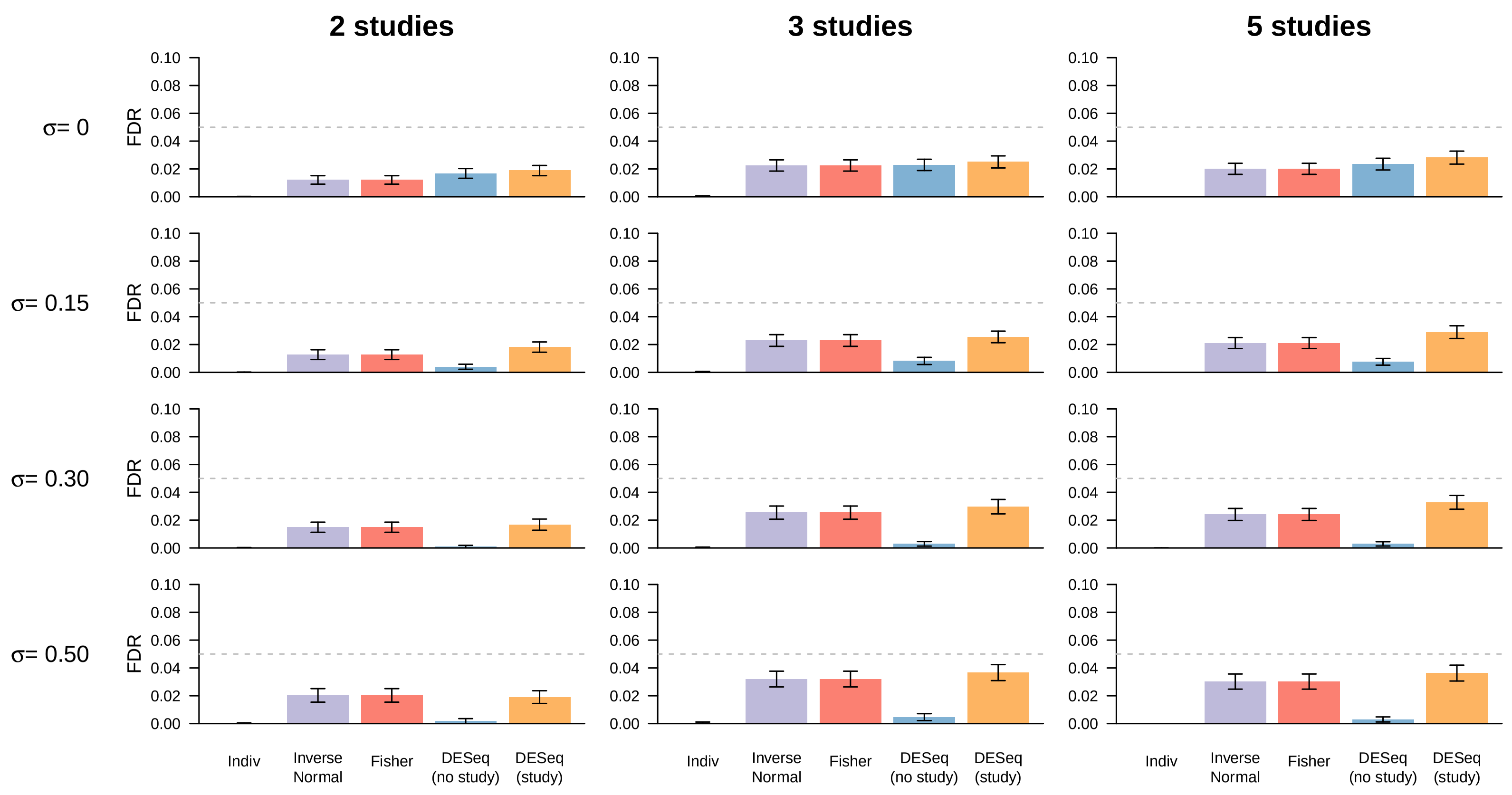

Supplementary Figure 2: False discovery rates (FDR) for all simulation settings. Each barplot represents the results of a particular setting, with columns corresponding (from left to right) to simulations including 2 studies, 3 studies, and 5 studies, and rows corresponding (from top to bottom) to simulations with inter-study variability set to $\sigma = 0$, $\sigma = 0.15$, $\sigma = 0.30$, and $\sigma = 0.50$ (no inter-study variability to large inter-study variability). Within each barplot, from left to right: Individual studies (green bars), Inverse Normal (purple bars), Fisher (red bars), DESeq with no study effect (blue bars), and DESeq with a fixed study effect (orange bars). Error bars represent one standard deviation.

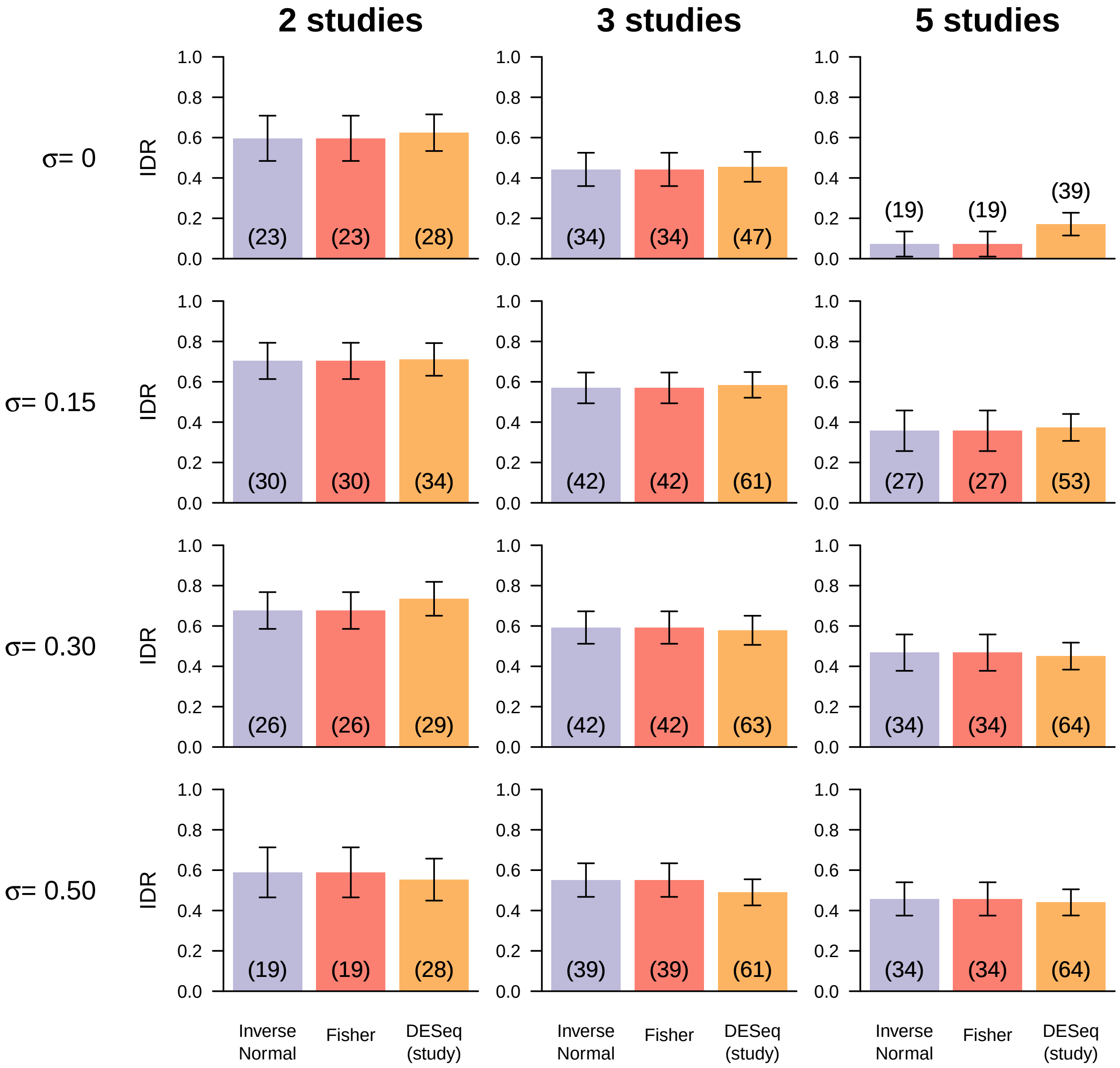

Supplementary Figure 3: Integration-driven discovery rates (IDR) for all simulation settings. The IDR represents the proportion of true positives among those uniquely identified by a given method (e.g., the Fisher approach) as compared to the individual per-study analyses. Each barplot represents the results of a particular setting, with columns corresponding (from left to right) to simulations including 2 studies, 3 studies, and 5 studies, and rows corresponding (from top to bottom) to simulations with inter-study variability set to $\sigma = 0$, $\sigma = 0.15$, $\sigma = 0.30$, and $\sigma = 0.50$ (no inter-study variability to large inter-study variability). Within each barplot, from left to right: Inverse Normal (purple bars), Fisher (red bars), and DESeq with a fixed study effect (orange bars). Error bars represent one standard deviation, and numbers in parentheses represent the mean total number of unique discoveries for each method as compared to the individual per-study analyses.

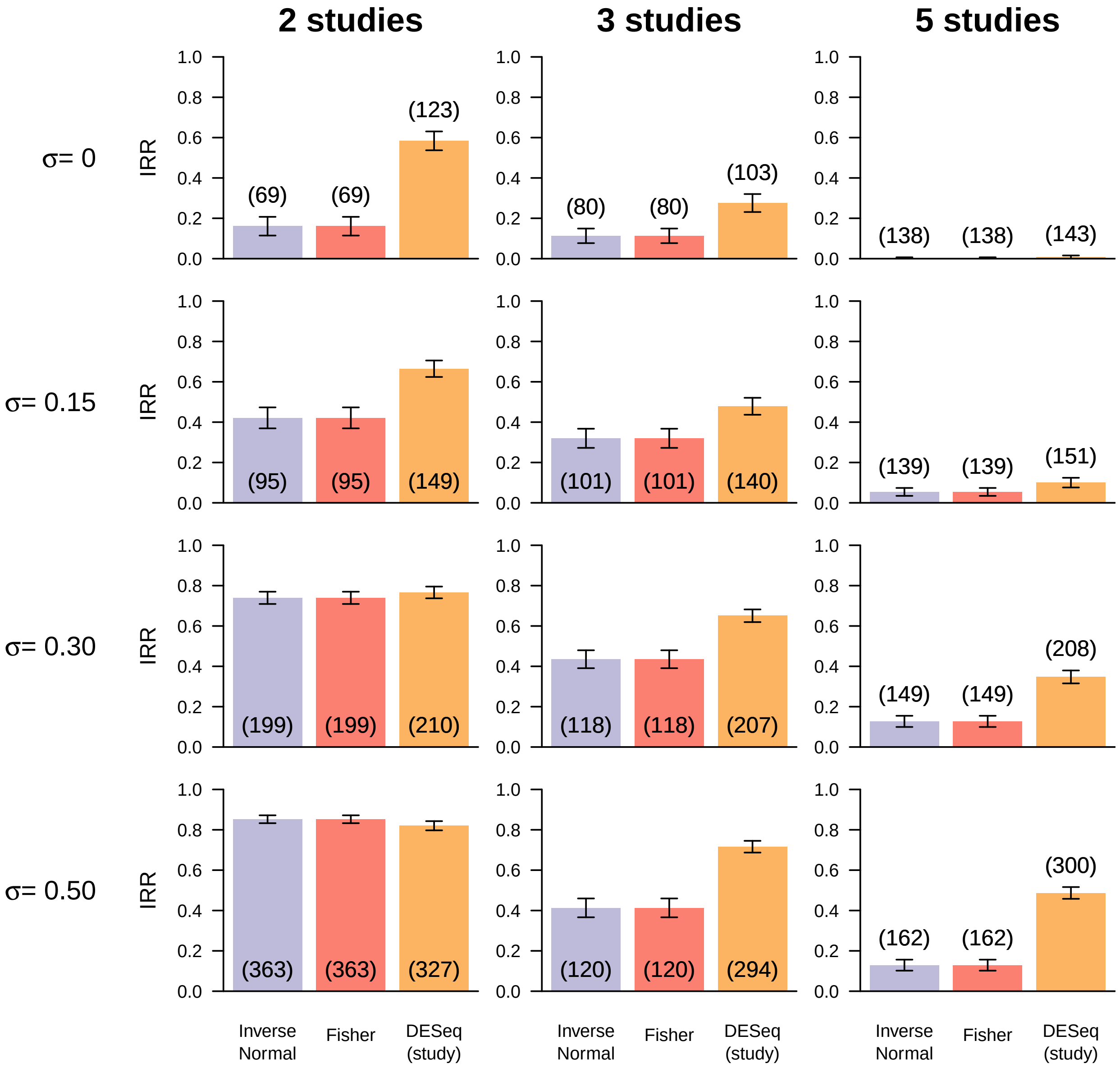

Supplementary Figure 4: Integration-driven revision rates (IRR) for all simulation settings. The IRR represents the proportion of true positives among those uniquely identified by the individual per-study analyses as compared to a given method (e.g., the Fisher approach). Each barplot represents the results of a particular setting, with columns corresponding (from left to right) to simulations including 2 studies, 3 studies, and 5 studies, and rows corresponding (from top to bottom) to simulations with inter-study variability set to $\sigma = 0$, $\sigma = 0.15$, $\sigma = 0.30$, and $\sigma = 0.50$ (no inter-study variability to large inter-study variability). Within each barplot, from left to right: Inverse Normal (purple bars), Fisher (red bars), and DESeq with a fixed study effect (orange bars). Error bars represent one standard deviation, and numbers in parentheses represent the mean total number of unique discoveries for the individual per-study analyses compared to each method.

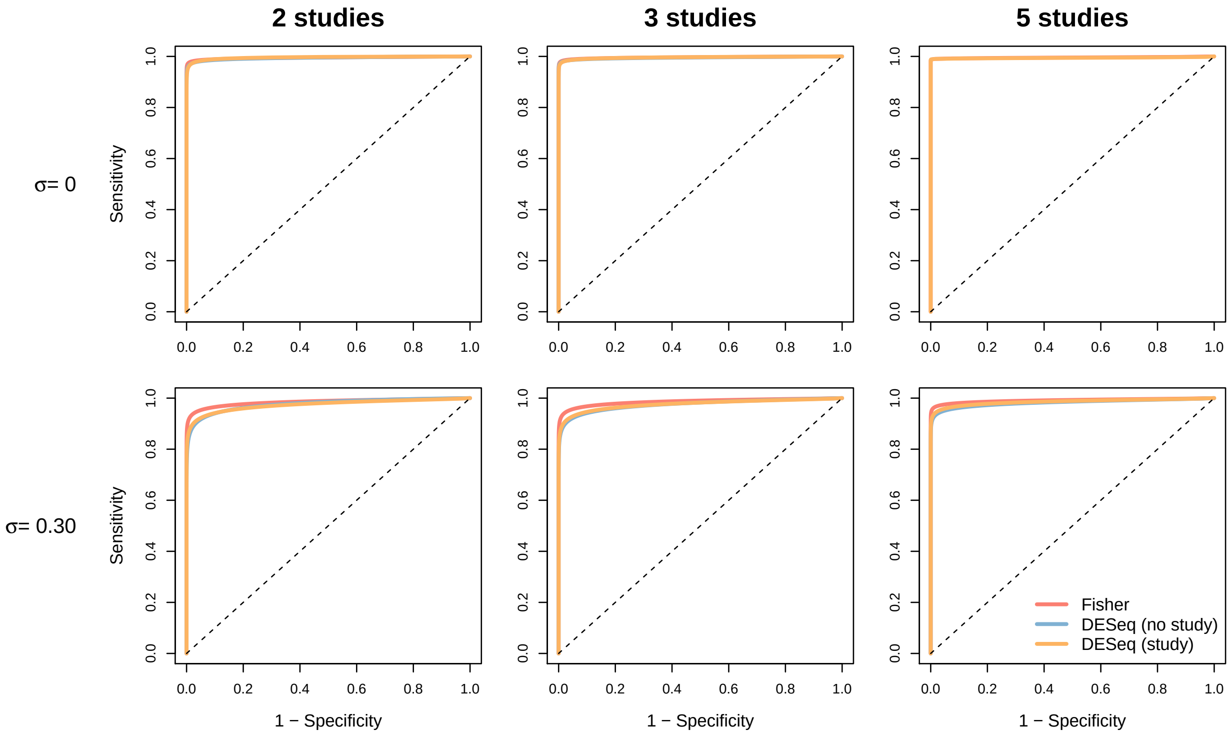

Supplementary Figure 5: Receiver Operating Characteristic (ROC) curves, for the simulation settings not demonstrated in the main paper. Each plot represents the results of a particular setting, with columns corresponding (from left to right) to simulations including 2 studies, 3 studies, and 5 studies, and rows corresponding (from top to bottom) to simulations with inter-study variability set to $\sigma = 0$ and $\sigma = 0.30$ (no inter-study variability to moderate inter-study variability). Within each plot: Fisher (red lines), DESeq with no fixed study effect (blue lines), and DESeq with a fixed study effect (orange lines).

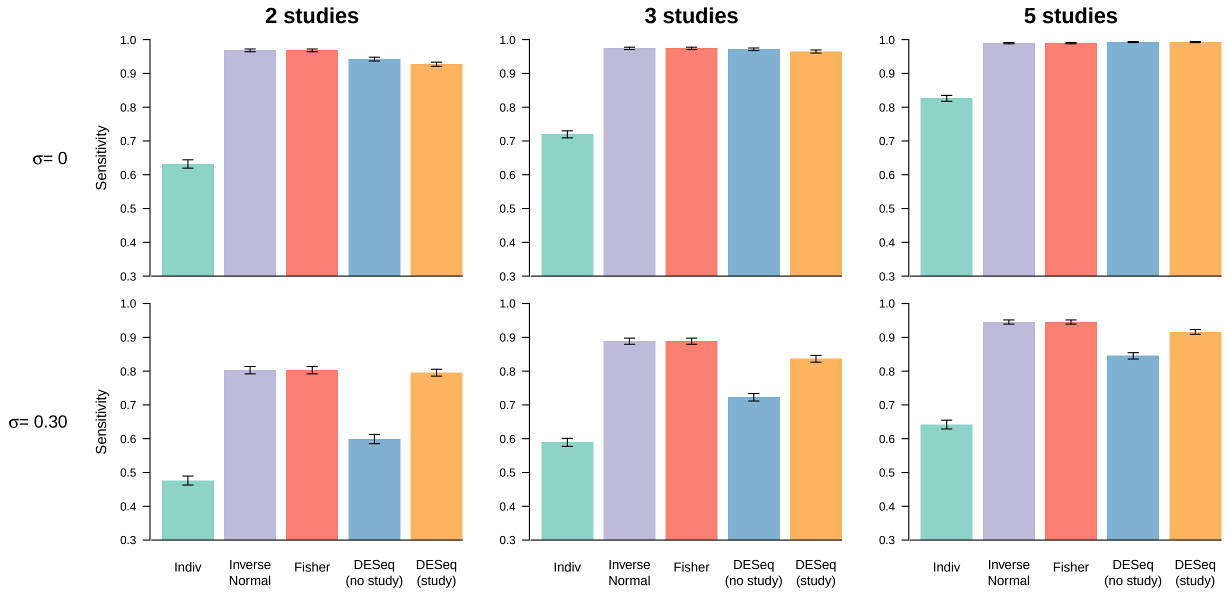

Supplementary Figure 6: Sensitivity, for the simulation settings not demonstrated in the main paper. Each barplot represents the results of a particular setting, with columns corresponding (from left to right) to simulations including 2 studies, 3 studies, and 5 studies, and rows corresponding (from top to bottom) to simulations with inter-study variability set to $\sigma = 0$ and $\sigma = 0.30$ (no inter-study variability to moderate inter-study variability). Within each barplot, from left to right: Individual per-study analyses (green bars), Inverse Normal (purple bars), Fisher (red bars), DESeq with no study effect (blue bars), and DESeq with a fixed study effect (orange bars).

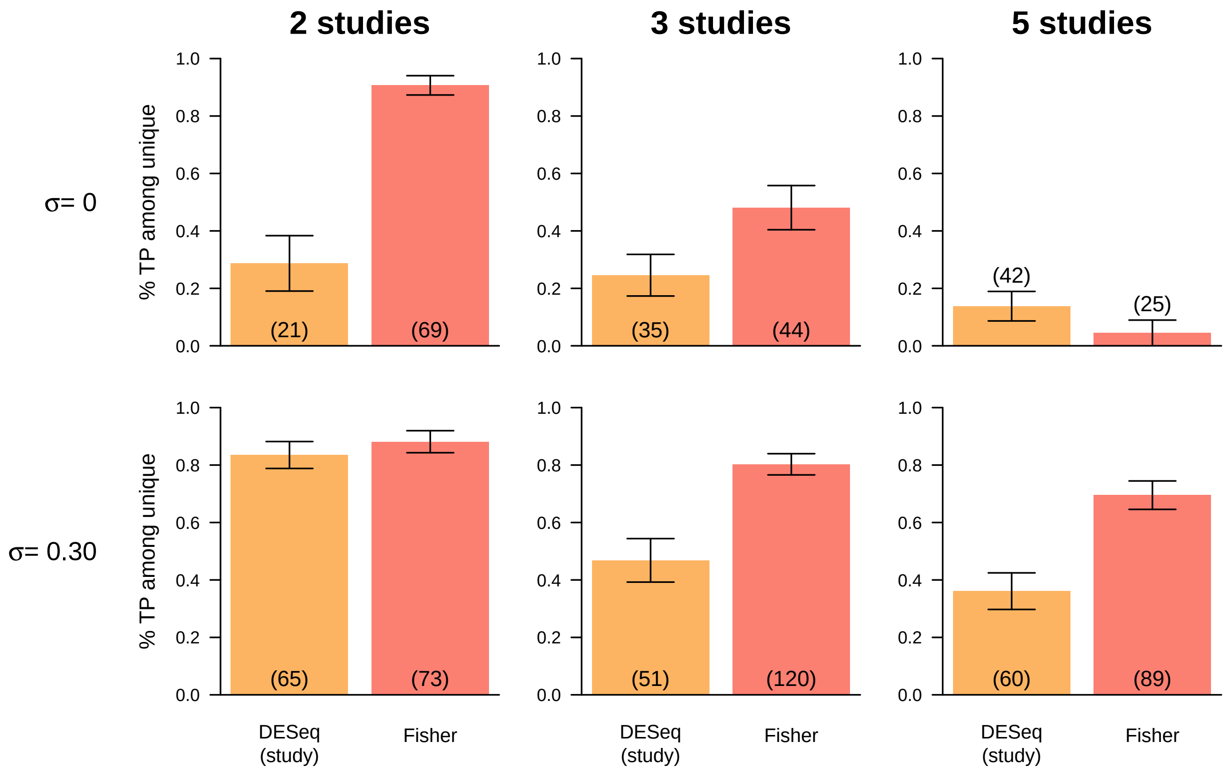

Supplementary Figure 7: Proportion of true positives among unique discoveries for DESeq with a fixed study effect (orange bars) and Fisher (red bars) for the simulation settings not demonstrated in the main paper. Each barplot represents the results of a particular setting, with columns corresponding (from left to right) to simulations including 2 studies, 3 studies, and 5 studies, and rows corresponding (from top to bottom) to simulations with inter-study variability set to $\sigma = 0$ and $\sigma = 0.30$ (no inter-study variability to moderate inter-study variability). Error bars represent one standard deviation, and numbers in parentheses represent the mean total number of unique discoveries for DESeq with study effect as compared to Fisher and vice versa, respectively.

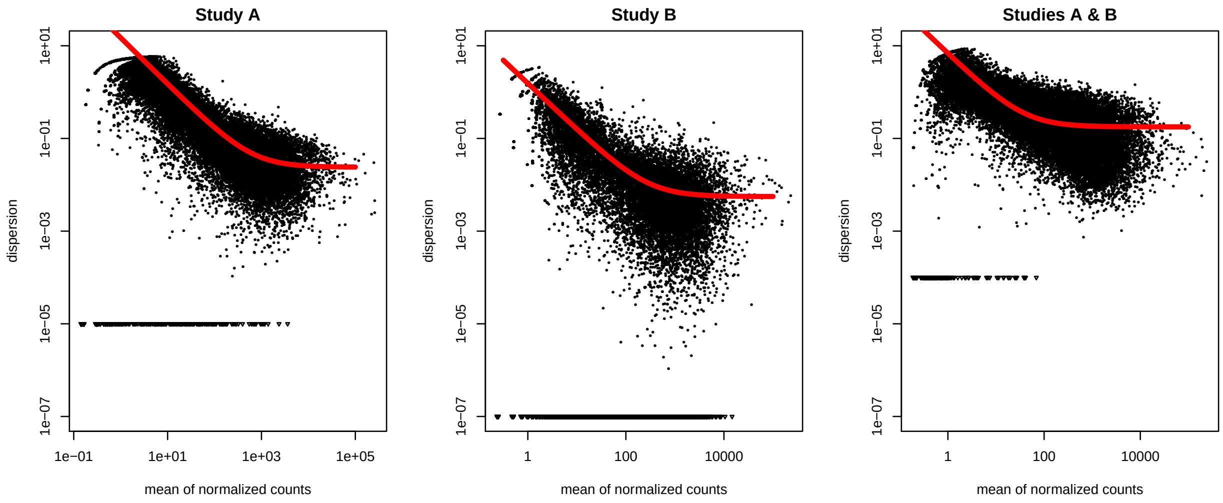

Supplementary Figure 8: Per-gene dispersion parameter estimates from *DESeq* plotted against mean normalized counts, calculated for Study A independently (left), Study B independently (middle), and Studies A and B together (right), with fitted gamma regressions superimposed as red lines.

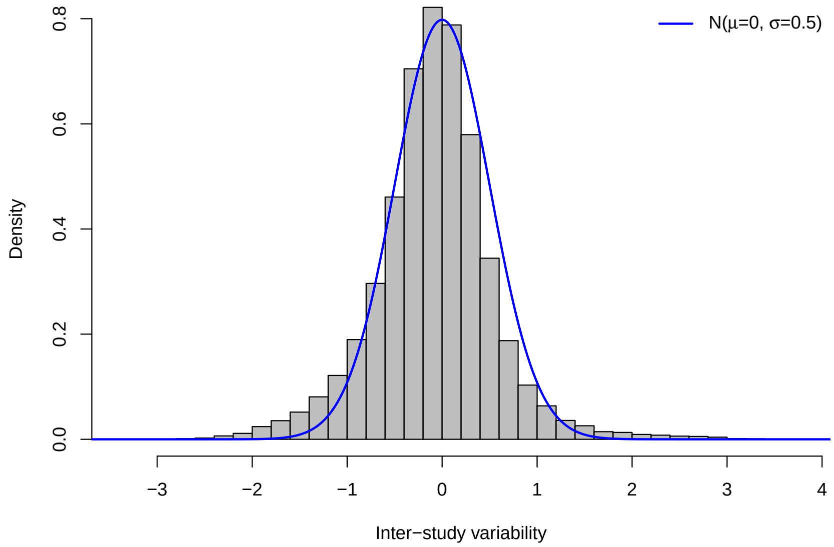

Supplementary Figure 9: Inter-study variability calculated on the Strub *et al.* (2011) data, with a $\mathcal{N}(0, \sigma = 0.5)$ density superimposed in blue. Inter-study variability was calculated as follows. We assume that the data follow a negative binomial distribution, $Y_{gcrs} \sim \text{NB}(\mu_{gcs}, \phi_{gs})$, where $\log(\mu_{gcs}) = w_{gc} + \varepsilon_{gcs}$, with $\varepsilon_{gcs} \sim \mathcal{N}(0, \sigma^2)$. To obtain an estimate for σ, the inter-study variability, we calculated the difference in log-transformed means in Study A from the log-transformed means in Study B for genes identified as non-differentially expressed in both of the individual per-study analyses. Under our previously stated assumption, the variance of this difference corresponds to $2\sigma^2$; as such, we divide the difference by 2 to obtain an estimate of σ^2.

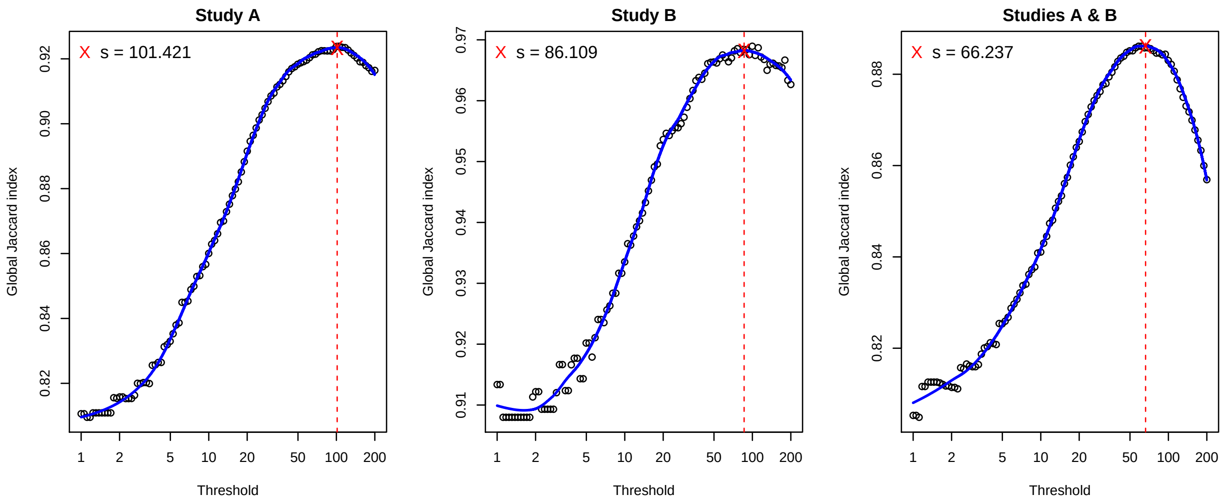

Supplementary Figure 10: Global Jaccard index calculated for a variety of threshold values for Study A independently (left), Study B independently (middle), and Studies A and B together (right), with a loess curve (blue line) superimposed and data-based threshold value indicated by the red cross and red dotted line.

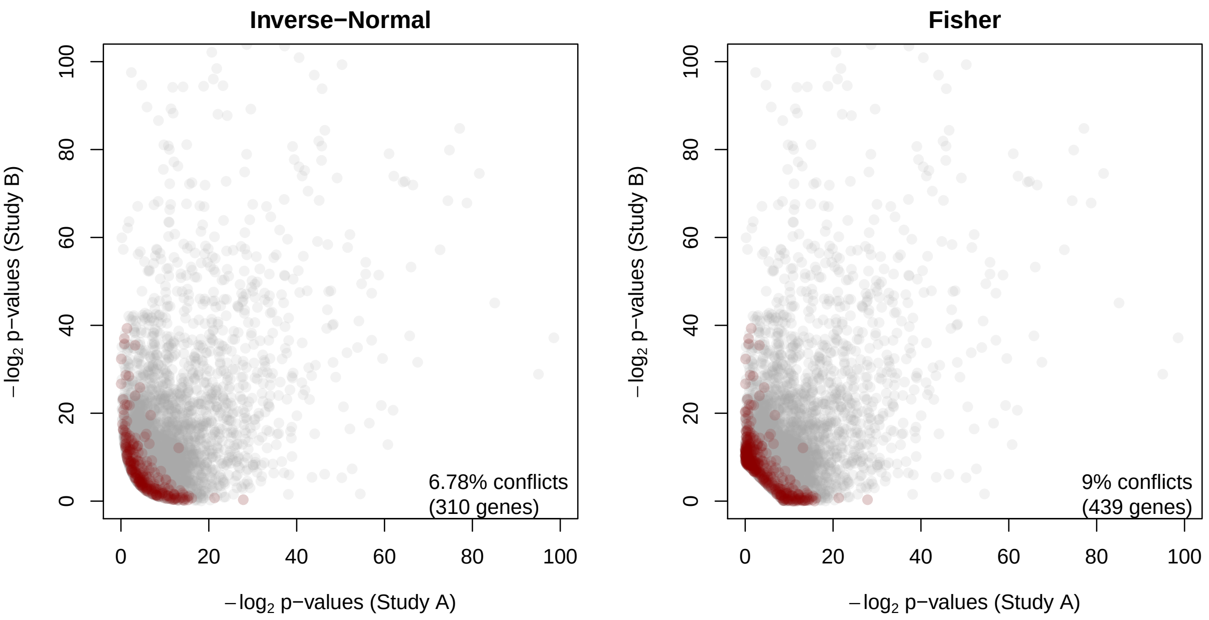

Supplementary Figure 11: -Log$_2$ raw p-values in Studies A and B for genes identified as differentially expressed using the Inverse normal (left) and Fisher (right) p-value combination methods, after adjusting combined p-values to control the false discovery rate at level $\alpha = 0.05$. Genes with differential expression conflicts are identified with red points.

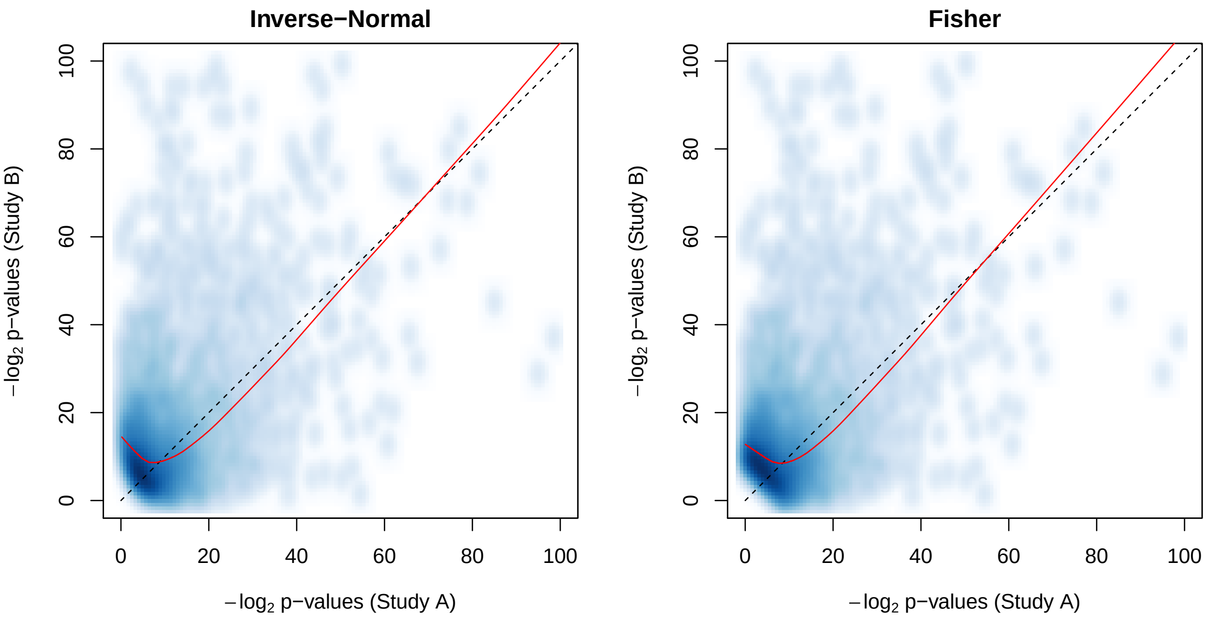

Supplementary Figure 12: -Log$_2$ raw p-values in Studies A and B for genes identified as differentially expressed using the Inverse normal (left) and Fisher (right) p-value combination methods, after adjusting combined p-values to control the false discovery rate at level $\alpha = 0.05$. The black dotted line represents the diagonal, and the red line represents a loess curve.

References

Andrea Rau, Mélina Gallopin, Gilles Celeux, and Florence Jaffrézic. Data-based filtering for replicated high-throughput transcriptome sequencing experiments. *(submitted)*, 2013.
