# Supplementary material for: Differential meta-analysis of RNA-seq data from multiple studies
Source: BMC Bioinformatics. 2014 Mar 29;15:91. doi: 10.1186/1471-2105-15-91 (PMC4021464; doi:10.1186/1471-2105-15-91)
Supplement: Additional file 2 — metaRNASeq vignette. This document contains a vignette describing in greater detail the metaRNASeq R package. It can also be obtained by running the following commands in the R console: > library(metaRNASeq) > vignette(“metaRNASeq”) [file 1471-2105-15-91-S2.pdf]

metaRNASeq: Differential meta-analysis of RNA-seq data

Guillemette Marot, Florence Jaffrézic, Andrea Rau

Modified: April 16, 2013. Compiled: June 13, 2013

Abstract

This vignette illustrates the use of the *metaRNASeq* package to combine data from multiple RNA-seq experiments. Based both on simulated and real publicly available data, it also explains the way the p-value data provided in the package have been obtained.

Contents

1 Introduction 1

2 Simulation study 2

3 Individual analyses of the two simulated data sets 3

4 Use of p-value combination techniques 5

5 Treatment of conflicts in differential expression 7

6 Session Info 9

1 Introduction

High-throughput sequencing (HTS) data, such as RNA-sequencing (RNA-seq) data, are increasingly used to conduct differential analyses, in which gene-by-gene statistical tests are performed in order to identify genes whose expression levels show systematic covariation with a particular condition, such as a treatment or phenotype of interest. Due to their large cost, however, only few biological replicates are often considered in each experiment leading to a low detection power of differentially expressed genes. For this reason, analyzing data arising from several experiments studying the same question can be a

useful way to increase detection power for the identification of differentially expressed genes.

The *metaRNASeq* package implements two p-value combination techniques (inverse normal and Fisher methods); see [3] for additional details. There are two fundamental assumptions behind the use of these p-value combination procedures: first, that p-values have been obtained the same way for each experiment (i.e., using the same model and test); and second, that they follow a uniform distribution under the null hypothesis. In this vignette, we illustrate these p-value combination techniques after obtaining p-values for differential expression in each individual experiment using the *DESeq* Bioconductor package [1]. Count data are simulated using the `sim.function` provided in the *metaRNASeq* package; see section 2 for additional detail.

2 Simulation study

To begin, we load the necessary packages and simulation parameters:

```
> library(metaRNASeq)
> library(DESeq)
> library(HTSFilter)
> data(param)
> dim(param)

[1] 26408     3

> data(dispFuncs)
```

These simulation parameters include the following information:

- `param`: Matrix of dimension (26408×3) containing mean expression in each of two conditions (here, labeled "condition 1" and "condition 2") and a logical vector indicating the presence or absence of differential expression for each of 26,408 genes

- `dispFuncs`: List of length 2, where each list is a vector containing the two estimated coefficients (α_0 and α_1) for the gamma-family generalized linear model (GLM) fit by *DESeq* (version 1.8.3) describing the mean-dispersion relationship for each of the two real datasets considered in [3]. These regressions represent the typical relationship between mean expression values μ and dispersions α in each dataset, where the coefficients α_0 and α_1 are found to parameterize the fit as $\alpha = \alpha_0 + \alpha_1/\mu$.

These parameters were calculated on real data sets from two human melanoma cell lines [5], corresponding to two different studies performed for the same cell line comparison, with two biological replicates per cell line in the first and three per cell line in the second. These data are presented in greater detail in [5] and [2], and are freely available in the Supplementary Materials of the latter.

Once parameters are loaded, we simulate data. We use the `set.seed` function to obtain reproducible results.

```
> set.seed(123)
> matsim <- sim.function(param = param, dispFuncs = dispFuncs)
> sim.conds <- colnames(matsim)
> rownames(matsim) <- paste("tag", 1:dim(matsim)[1],sep="")
> dim(matsim)

[1] 26408    16
```

The simulated matrix data contains 26, 408 genes and 4 replicates per condition per study. It is possible to change the number of replicates in each study using either the `nrep` argument or the `classes` argument. Using `nrep` simulates the same number of replicates per condition per study. In order to simulate an unbalanced design, the `classes` argument may be used. For example, setting

```
classes = list(c(1,2,1,1,2,1,1,2),c(1,1,1,2,2,2,2))
```

leads to 5 and 3 replicates in each condition for the first study, and 3 and 4 replicates in each condition in the second.

3 Individual analyses of the two simulated data sets

Before performing a combination of p-values from each study, it is necessary to perform a differential analysis of the individual studies (using the same method). In the following example, we make use of the *DESeq* package to obtain p-values for differential analyses of each study independently; however, we note that other differential analysis methods (e.g., *edgeR* or *baySeq*) could be used prior to the meta analysis.

Genes with very low values of expression often lead to an enrichment of p-values close to 1 as they take on discrete values; as such genes are unlikely to display evidence for differential expression, it has been proposed to apply *independent filtering* to filter these genes [4]. In addition, the application of such a filter typically removes those genes contributing to a peak of p-values close to 1, leading to a distribution of p-values under the null hypothesis more closely following a uniform distribution. As the proposed p-value combination techniques rely on this assumption, it is thus necessary to independently filter genes with very low read counts. For this purpose, we recommand the use of the *HTSFilter* package, see [4] for more details; note that we apply the filter in *HTSFilter* to each study individually after estimating library sizes and per-gene dispersion parameters.

Once the data are filtered, we use the *DESeq* package to perform differential analyses of each of the two individual datasets. The following function `resDESeq1study` is a wrapper of the main functions of the data filter in *HTSFilter* and differential analysis in *DESeq*, selecting the appropriate columns in the simulated data set for each study.

The following two steps could be replaced by direct uses of the *HTSFilter* and *DESeq* packages and concatenation of results in one list (see `resDESeq.alt`).

```
> resDESeq1study <- function(studyname, alldata, cond1totest="cond1",
+     cond2totest="cond2", fitType = "parametric") {
+   study <- alldata[,grep(studyname,colnames(alldata))]
+   studyconds <- gsub(studyname,"",colnames(study))
+   colnames(study) <- paste(studyconds,1:dim(study)[2],sep=".")
+   cds <- newCountDataSet(study, studyconds)
+   cds <- estimateSizeFactors(cds)
+   cds <- estimateDispersions(cds, method="pooled", fitType=fitType)
+   ## Filter using Jaccard index for each study
+   filter <- HTSFilter(cds, plot=FALSE)
+   cds.filter <- filter$filteredData
+   on.index <- which(filter$on == 1)
+   cat("# genes passing filter", studyname, ":", dim(cds.filter)[1], "\n")
+   res <- as.data.frame(matrix(NA, nrow = nrow(cds), ncol=ncol(cds)))
+   nbT <- nbinomTest(cds.filter, cond1totest, cond2totest)
+   colnames(res) <- colnames(nbT)
+   res[on.index,] <- nbT
+   res
+ }
```

The wrapper can be applied simultaneously to the two studies with the use of the function `lapply`:

```
> studies <- c("study1", "study2")
> resDESeq <- lapply(studies,
+   FUN=function(x) resDESeq1study(x, alldata=matsim))

# genes passing filter study1 : 14044
# genes passing filter study2 : 13839
```

Note that `resDESeq` can be created directly from two or more *DEseq* results called `res.study1`, `res.study2`, . . . :

```
> #alternative approach
> #resDESeq.alt <- list(res.study1,res.study2)
```

Since only p-values are necessary to perform meta-analysis, we keep them in lists called `rawpval` for raw p-values and `adjpval` for p-values adjusted to correct for multiple testing (e.g., to control the false discovery rate at 5% using the Benjamini-Hochberg method).

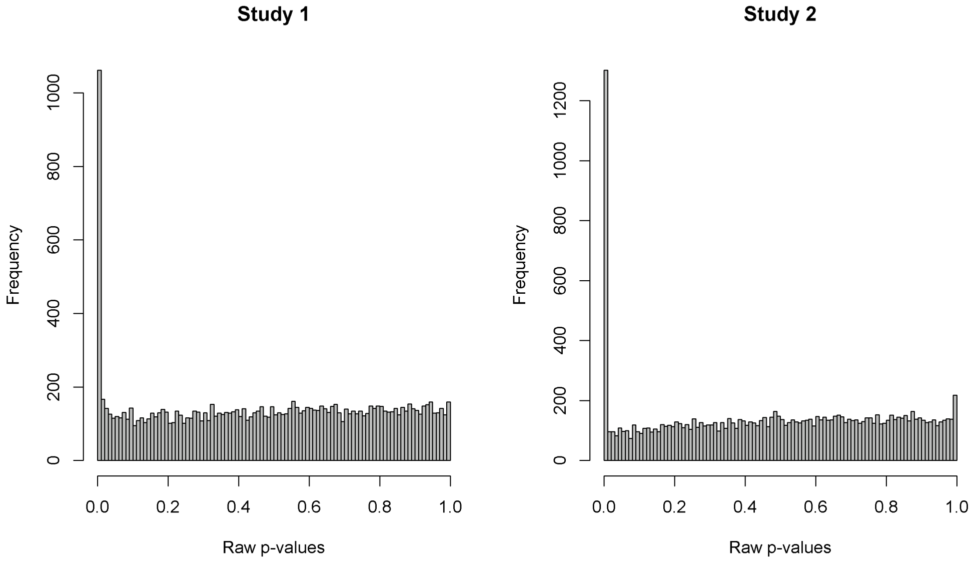

Figure 1: Histograms of raw p-values for each of the individual differential analyses performed using the *DESeq* package.

```
> rawpval <- lapply(resDESeq, FUN=function(res) res$pval)
> adjpval <- lapply(resDESeq, FUN=function(res) res$padj)
> DE <- mapply(adjpval, FUN=function(x) ifelse(x <= 0.05, 1, 0))
> colnames(DE)=paste("DE",studies,sep=".")
```

DE returns a matrix with 1 for genes identified as differentially expressed and 0 otherwise (one column per study). To confirm that the raw p-values under the null hypothesis are roughly uniformly distributed, we may also inspect histograms of the raw p-values from each of the individual differential analyses (see Figure 1):

```
> par(mfrow = c(1,2))
> hist(rawpval[[1]], breaks=100, col="grey", main="Study 1",
+   xlab="Raw p-values")
> hist(rawpval[[2]], breaks=100, col="grey", main="Study 2",
+   xlab="Raw p-values")
```

4 Use of p-value combination techniques

The code in this section may be used independently from the previous section if p-values from each study have been obtained using the same differential analysis test between

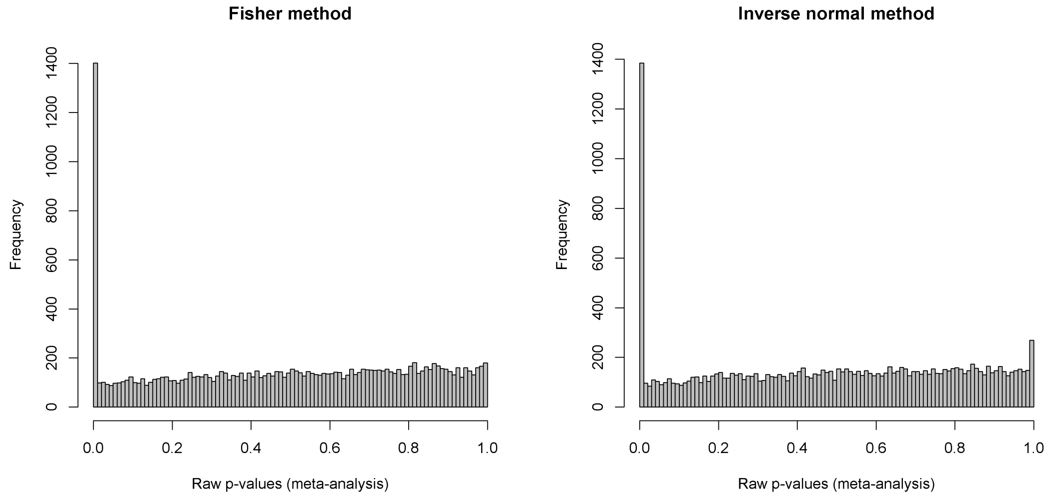

Figure 2: (Left) Histogram of raw p-values obtained after a meta-analysis of all studies, with p-value combination performed using the Fisher method. (Right) Histogram of raw p-values obtained after a meta-analysis of all studies, with p-value combination performed using the inverse normal method.

the different studies. Vectors of p-values must have the same length; `rawpval` is a list (or data.frame) containing the vectors of raw p-values obtained from the individual differential analyses of each study.

The p-value combination using the Fisher method may be performed with the `fishercomb` function, and the subsequent p-values obtained from the meta-analysis may be examined (Figure 2, left):

```
> fishcomb <- fishercomb(rawpval, BHth = 0.05)
> hist(fishcomb$rawpval, breaks=100, col="grey", main="Fisher method",
+   xlab = "Raw p-values (meta-analysis)")
```

The use of the inverse normal combination technique requires the choice of a weight for each study. In this example, we choose `nrep=8`, since 8 replicates had been simulated in each study. As before, we may examine a histogram of the subsequent p-values obtained from the meta-analysis (Figure 2, right).

```
> invnormcomb <- invnorm(rawpval,nrep=c(8,8), BHth = 0.05)
> hist(invnormcomb$rawpval, breaks=100, col="grey",
+   main="Inverse normal method",
+   xlab = "Raw p-values (meta-analysis)")
```

Finally, we suggest summarizing the results of the individual differential analyses as well as the differential meta-analysis (using the Fisher and inverse normal methods) in a data.frame:

```
> DEresults <- data.frame(DE,
+   "DE.fishercomb"=ifelse(fishcomb$adjpval<=0.05,1,0),
+   "DE.invnorm"=ifelse(invnormcomb$adjpval<=0.05,1,0))
> head(DEresults)

  DE.study1 DE.study2 DE.fishercomb DE.invnorm
1         0         0             0          0
2         0         0             0          0
3         0         0             0          0
4         1         1             1          1
5        NA        NA            NA         NA
6         0         0             0          0
```

5 Treatment of conflicts in differential expression

As pointed out in [3], it is not possible to directly avoid conflicts between over- and under- expressed genes in separate studies that appear in differential meta-analyses of RNA-seq data. We thus advise checking that individual studies identify differential expression in the same direction (i.e., if in one study, a gene is identified as differentially over-expressed in condition 1 as compared to condition 2, it should not be identified as under-expressed in condition 1 as compared to condition 2 in a second study). Genes displaying contradictory differential expression in separate studies should be removed from the list of genes identified as differentially expressed via meta-analysis.

We build a matrix `FC` gathering all fold changes from individual studies.

```
> FC1study <- function(studyname, alldata=matsim, cond1totest="cond1",
+     cond2totest="cond2") {
+   study <- alldata[,grep(studyname,colnames(alldata))]
+   studyconds <- gsub(studyname,"",colnames(study))
+   colnames(study) <- paste(studyconds,1:dim(study)[2],sep=".")
+   cds <- newCountDataSet(study, studyconds)
+   cds <- estimateSizeFactors(cds)
+   ## Calculate normalized base means using DESeq functions
+   bm1 <- getBaseMeansAndVariances(counts(cds[,which(studyconds=="cond1")]),
+          sizeFactors(cds)[which(studyconds=="cond1")])$baseMean
+   bm2 <- getBaseMeansAndVariances(counts(cds[,which(studyconds=="cond2")]),
+          sizeFactors(cds)[which(studyconds=="cond2")])$baseMean
+   FC <- log2(bm2 / bm1)
+   names(FC) <- rownames(study)
+   FC
+ }
> FC <- mapply(FC1study, studyname=c("study1", "study2"))
```

```
> sumsigns <- apply(sign(FC),1,sum)
> commonsgnFC <- ifelse(abs(sumsigns)==dim(FC)[2], sign(sumsigns),0)
```

The vector `commonsgnFC` will return a value of 1 if the gene has a positive $\log_2$ fold change in all studies, -1 if the gene has a negative $\log_2$ fold change in all studies, and 0 if contradictory $\log_2$ fold changes are observed across studies (i.e., positive in one and negative in the other). By examining the elements of `commonsgnFC`, it is thus possible to identify genes displaying contradictory differential expression among studies.

```
> unionDE <- unique(c(fishcomb$DEindices,invnormcomb$DEindices))
> FC.selecDE <- data.frame(DEresults[unionDE,],FC[unionDE,],
+   signFC=commonsgnFC[unionDE], DE=param$DE[unionDE])
> keepDE <- FC.selecDE[which(abs(FC.selecDE$signFC)==1),]
> conflictDE <- FC.selecDE[which(FC.selecDE$signFC == 0),]
> dim(FC.selecDE)

[1] 1356    8

> dim(keepDE)

[1] 1183    8

> dim(conflictDE)

[1] 173   8

> head(keepDE)

   DE.study1 DE.study2 DE.fishercomb DE.invnorm     study1
4          1         1             1          1  1.3953474
11         1         1             1          1 -0.9995495
22         1         1             1          1 -1.1846661
36         1         1             1          1 -3.0703584
55         0         1             1          1 -0.4229661
59         1         1             1          1  1.1465223
       study2 signFC   DE
4   2.0827094      1 TRUE
11 -0.5666571     -1 TRUE
22 -0.9829683     -1 TRUE
36 -2.8604070     -1 TRUE
55 -1.0557700     -1 TRUE
59  1.3520322      1 TRUE
```

Note that out of the 173 conflicts, 147 represented genes that were simulated to be truly differentially expressed.

```
> table(conflictDE$DE)

FALSE  TRUE
   26   147
```

6 Session Info

```
> sessionInfo()

R version 2.15.2 (2012-10-26)
Platform: x86_64-w64-mingw32/x64 (64-bit)

locale:
[1] LC_COLLATE=French_France.1252
[2] LC_CTYPE=French_France.1252
[3] LC_MONETARY=French_France.1252
[4] LC_NUMERIC=C
[5] LC_TIME=French_France.1252

attached base packages:
[1] stats     graphics  grDevices utils     datasets
[6] methods   base

other attached packages:
 [1] SweaveListingUtils_0.5.5 startupmsg_0.7.2
 [3] HTSFilter_0.99.6         edgeR_3.0.6
 [5] limma_3.14.3             DESeq_1.10.1
 [7] lattice_0.20-10          locfit_1.5-8
 [9] Biobase_2.18.0           BiocGenerics_0.4.0
[11] metaRNASeq_0.2

loaded via a namespace (and not attached):
 [1] annotate_1.36.0      AnnotationDbi_1.20.3
 [3] DBI_0.2-5            genefilter_1.40.0
 [5] geneplotter_1.36.0   grid_2.15.2
 [7] IRanges_1.16.4       parallel_2.15.2
 [9] RColorBrewer_1.0-5   RSQLite_0.11.2
[11] splines_2.15.2       stats4_2.15.2
[13] survival_2.37-2      tools_2.15.2
[15] XML_3.95-0.1         xtable_1.7-0
```

References

[1] S. Anders and W. Huber. Differential expression analysis for sequence count data. *Genome Biology*, 11(R106):1–28, 2010.

[2] M.-A. Dillies, A. Rau, J. Aubert, C. Hennequet-Antier, M. Jeanmougin, N. Servant, C. Keime, G. Marot, D. Castel, J. Estelle, G. Guernec, B. Jagla, L. Jouneau, D. Laloë,

C. Le Gall, B. Schaëffer, S. Le Crom, and F. Jaffrézic. A comprehensive evaluation of normalization methods for Illumina high-throughput RNA sequencing data analysis. *Briefings in Bioinformatics*, 2012. doi: 10.1093/bib/bbs046.

[3] A. Rau, G. Marot, and F. Jaffrézic. Differential meta-analysis of RNA-seq data from multiple studies. *(submitted)*, 2013.

[4] Andrea Rau, Mélina Gallopin, Gilles Celeux, and Florence Jaffrézic. Data-based filtering for replicated high-throughput transcriptome sequencing experiments. *(submitted)*, 2013.

[5] T. Strub, S. Giuliano, T. Ye, C. Bonet, C. Keime, D. Kobi, S. Le Gras, M. Cormont, R. Ballotti, C. Bertolotto, and I. Davidson. Essential role of microphthalmia transcription factor for DNA replication, mitosis and genomic stability in melanoma. *Oncogene*, 30:2319–2332, 2011.
